# Activation and inhibition of the sweet taste receptor TAS1R2-TAS1R3 differentially affect glucose tolerance in humans

**DOI:** 10.1371/journal.pone.0298239

**Published:** 2024-05-01

**Authors:** Matthew C. Kochem, Emily C. Hanselman, Paul A. S. Breslin

**Affiliations:** 1 Department of Nutritional Sciences, Rutgers University, New Brunswick, NJ, United States of America; 2 Monell Chemical Senses Center, Philadelphia, PA, United States of America; Federal University of Pelotas: Universidade Federal de Pelotas, BRAZIL

## Abstract

The sweet taste receptor, TAS1R2-TAS1R3, is expressed in taste bud cells, where it conveys sweetness, and also in intestinal enteroendocrine cells, where it may facilitate glucose absorption and assimilation. In the present study, our objective was to determine whether TAS1R2-TAS1R3 influences glucose metabolism bidirectionally via hyperactivation with 5 mM sucralose (n = 12) and inhibition with 2 mM sodium lactisole (n = 10) in mixture with 75 g glucose loads during oral glucose tolerance tests (OGTTs) in healthy humans. Plasma glucose, insulin, and glucagon were measured before, during, and after OGTTs up to 120 minutes post-prandially. We also assessed individual participants’ sweet taste responses to sucralose and their sensitivities to lactisole sweetness inhibition. The addition of sucralose to glucose elevated plasma insulin responses to the OGTT (F(1, 11) = 4.55, p = 0.056). Sucralose sweetness ratings were correlated with early increases in plasma glucose (R^2^ = 0.41, p<0.05), as well as increases in plasma insulin (R^2^ = 0.38, p<0.05) when sucralose was added to the OGTT (15 minute AUC). Sensitivity to lactisole sweetness inhibition was correlated with decreased plasma glucose (R^2^ = 0.84, p<0.01) when lactisole was added to the OGTT over the whole test (120 minute AUC). In summary, stimulation and inhibition of the TAS1R2-TAS1R3 receptor demonstrates that TAS1R2-TAS1R3 helps regulate glucose metabolism in humans and may have translational implications for metabolic disease risk.

## Introduction

The canonical carbohydrate regulatory pathway involves pancreatic recognition of plasma glucose via glucose transport, oxidation, and ATP production (the metabolic signaling pathway), resulting in the release of insulin to regulate glycemia. Several studies have suggested that the TAS1R2-TAS1R3 receptor contributes to the regulation of carbohydrate metabolism. TAS1R2-TAS1R3 has been implicated in several carbohydrate regulatory processes, including: cephalic phase pre-absorptive insulin release [1], enteroendocrine responses of GLP-1 [2,3], upregulation and translocation of SGLT1 and GLUT2 transporters in the intestine [4–7] and direct stimulation of insulin from the pancreas [8–10]. The TAS1R2-TAS1R3 receptor is a heteromeric receptor of the seven transmembrane family, Class C and was originally identified as the chief transducer of sweet taste perception via functional expression studies and mouse knock-out data [11,12], and is activated by several mono- and disaccharides. It can also be stimulated by certain amino acids, metal salts and high potency sweeteners (HPS) [13] and is inhibited by sodium lactisole, an inverse agonist which binds the transmembrane domain of TAS1R3 [14,15].

In addition to its role in oral taste transduction, TAS1R2-TAS1R3 is expressed in metabolic regulatory organs, including the intestine and pancreas. In these tissues, TAS1R2-TAS1R3 serves as a sugar sensor to facilitate glucose absorption and assimilation. For example, TAS1R2-TAS1R3 is expressed on the surface of enteroendocrine L-cells, which secrete GLP-1 upon exposure to HPS [2,16]. Consequently, TAS1R2-TAS1R3 may influence glucose clearance from blood via enhancement of glucose stimulated insulin secretion. This effect is blocked by lactisole, a TAS1R2-TAS1R3 inverse agonist [16]. In *ex vivo* mouse models, intestinal perfusion with sucralose, a HPS, stimulates GLP-1 and GLP-2 secretion [3]. In knockout (KO) mouse models, *Tas1r3* ablation abolishes the GLP-1 response to the presence of glucose in the intestine [2]. Furthermore, TAS1R2-TAS1R3 stimulation may enhance glucose absorption. HPS stimulation of the intestine upregulates intestinal glucose transporters in animal models, specifically luminal SGLT1 expression increases in mice in a *Tas1r3*-dependent manner [4]. Acute intestinal perfusion with HPS may also increase apical translocation of GLUT2 in mice within minutes [5,6].

Pancreatic beta cells also respond to HPS *in vitro*. TAS1R2-TAS1R3 is expressed in pancreatic beta cells in humans and mice [8,17]. *In vitro*, human and mouse beta cells secrete insulin when exposed to sweeteners and this effect is blocked by sweet taste inhibitors [8–10]. *Tas1r2* knockout mice display reduced plasma glucose responses to glucose loads [6,18]. Strikingly, *Tas1r2* knockout animals are partially protected from metabolic disturbances associated with diet-induced obesity, including hyperinsulinemia [19]. In healthy humans, when lactisole is added to an oral glucose tolerance test to inhibit TAS1R2-TAS1R3 activity, plasma insulin increases compared to the control and plasma glucose is unaffected [20].

In contrast to mouse studies, human studies examining the metabolic effects of HPS consumption have yielded mixed results [21]. HPS consumption 10–30 minutes prior to consumption of 75 g glucose sometimes shows effects of elevated insulin [22–24] and sometimes does not [25–29]. HPS consumption in the absence of glucose has not been observed to affect plasma GLP-1, insulin, or glucose [30–32]. It is difficult to draw conclusions from the extant literature because previous studies vary in terms of which HPS was used, the HPS doses, and the timing of delivery relative to physiological analysis. Previous studies have been designed using doses of HPS that would typically be found in one serving of diet soda. Although this design lends real-world generalizability, it does not clearly test nor establish the basic function of TAS1R2-TAS1R3 in human regulatory physiology.

We reasoned that in order to assess the contribution of TAS1R2-TAS1R3 to orally-consumed glucose tolerance, stimuli should be designed to hyperstimulate the sweet receptor. Thus, HPS should be used at a higher dose and presented directly in mixture with glucose. This approach allows us to establish whether TAS1R2-TAS1R3 participates in plasma glucose regulation in humans. To capture the full range of receptor activity, the effect of TAS1R2-TAS1R3 inhibition was examined as well. Therefore, to test the carbohydrate regulatory capacity of TAS1R2-TAS1R3, we sought to bidirectionally hyperstimulate the receptor with sucralose and inhibit the receptor with lactisole. We hypothesized that in a standard oral glucose tolerance test, hyperstimulation with sucralose would enhance or hasten postprandial glucose and insulin responses, whereas inhibition with lactisole would suppress or delay postprandial glucose and require increased insulin responses due to reduced expectation of incoming glucose. Furthermore, we hypothesized that individual differences in metabolic responses to sucralose and lactisole would mirror individuals’ sweet taste ratings, which may serve as an indicator of functional differences in the TAS1R2-TAS1R3 transduction pathway irrespective of the metabolic tissue in which it is expressed.

In the present study, we determined whether TAS1R2-TAS1R3 hyperactivation and inhibition concomitant with glucose loads would modulate glucose tolerance in humans. In the first study, we conducted oral glucose tolerance tests in twelve healthy participants with and without the addition of 5 mM sucralose. In a second study, we conducted oral glucose tolerance tests in ten healthy participants with and without the addition of 2 mM lactisole. Following the oral glucose tolerance tests, we assessed individual differences of the sweet taste perceptual system to activation by sucralose and inhibition by lactisole to determine the efficacy of our TAS1R2-TAS1R3 treatments in each participant. This assessment enabled us to examine the functional relationships between perceptual and physiological responses, for example, we conducted a sub-analysis of those most responsive to sucralose and lactisole.

## Materials and methods

### Participants

There were 19 participants total in both studies. Participants were recruited between the dates of 11/12/2012 to 10/06/2014 through flyers posted on the Rutgers University campus. Participants provided written informed consent and were paid for their participation. All procedures in this study were approved by the institutional review board at Rutgers University, protocol number IRB 16-105Mc. This protocol complied with the Declaration of Helsinki for Medical Research involving Human Subjects. The study was also registered at ClinicalTrials.gov, identifier NCT05900193.

28 participants were screened with a medical and dietary questionnaire used in previous studies. Participants who reported consuming more than one serving of artificially sweetened beverages or snacks per day were excluded. Participants with diseases (e.g. metabolic syndrome and diabetes) and medications that may affect taste, digestion and absorption (e.g. anti-hypertensives, antibiotics, insulin, metformin, SGLT2 Inhibitors, sulfonylureas) were also excluded. Participants with BMI>30 kg/m^2^ were excluded. Based on these criteria, 9 individuals were excluded from participating. Of the 19 participants, nine took part in the sucralose experiment only, seven took part in the lactisole experiment only and three participants took part in both experiments. Participant traits are summarized in Table 1. There were no significant differences in any traits between participants in either experiment.

**Table 1:** Participant traits.

	Sucralose Study	Lactisole Study	P value
**Number of participants**	12	10	-
**Age (years)**	26.2 +/- 1.2	25.6 +/- 1.1	0.74
**Sex (% female)**	67%	50%	0.45
**BMI (kg/m** ^**2**^ **)**	23.0 +/- 0.9	23.3 +/- 0.7	0.77
**Fasting glucose (mg/dl)**	93.7 +/- 2.8	91.8 +/- 2.4	0.63

This table depicts means +/- standard error for each phenotype group.

### Glucose tolerance tests

**Fig 1:**
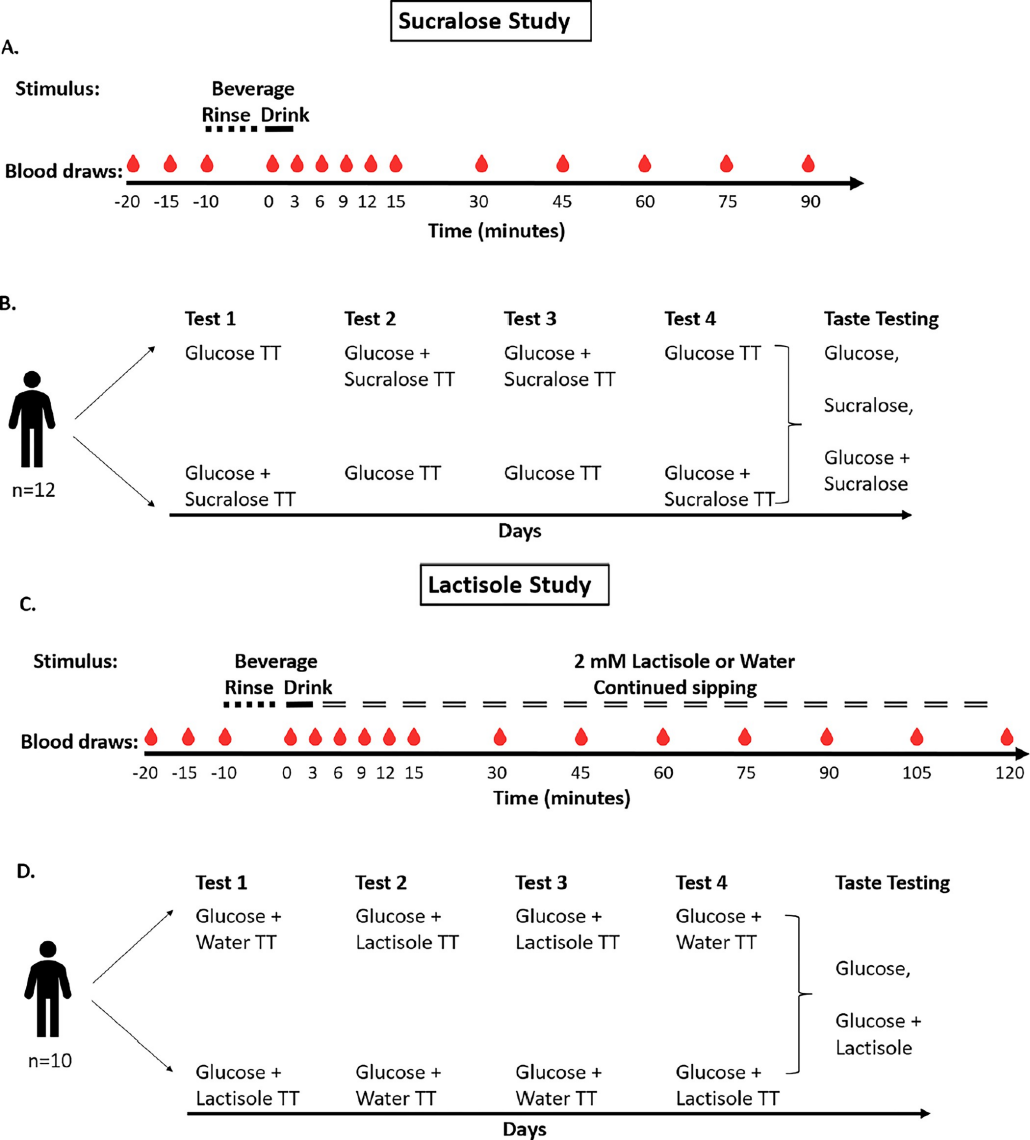


Participants were tested in a randomized, single blind, crossover design (Fig 1). Participants were tested on four occasions, spaced one week apart. Participants were instructed to fast after midnight before coming to the laboratory. All participants were tested in the morning at 9 am. Participants were also instructed to abstain from structured exercise for the 48 hours preceding each test. Blood samples were collected using an indwelling catheter inserted into an antecubital vein. Blood samples were collected in chilled EDTA plasma tubes. Samples were centrifuged (4 degrees, 15 minutes, 1300 RCF) and plasma was separated and stored at -80 degrees for later analysis. Plasma insulin and glucagon were measured using a double-antibody radioimmunoassay (EMD Millipore, Billerica MA). Glucose was measured spectrophotometrically using an auto-analyzer (Roche). These measurements were made by a core facility of the University of Pennsylvania Institute for Diabetes, Obesity, and Metabolism.

12 participants were randomized in a crossover design to consume either a 1.39 M glucose solution (75 g glucose dissolved in 300 mL water) or a 1.39 M glucose solution with added 5 mM sucralose (75 g glucose + 0.6 g sucralose in 300 mL water) first, and then tested with the other solution at least one week later (Fig 1B). This procedure was repeated in the reverse order following an ABBA design. Our concentration of 5 mM sucralose is approximately 12 times higher than levels used in diet soda (~0.4 mM) and is rated as strongly sweet on average on a Labeled Magnitude Scale. We selected this concentration of sucralose to ensure that we were testing the hypothesis that TAS1R2-TAS1R3 activation regulates glucose metabolism. Baseline blood samples were collected at -20, -15, and -10 minutes (Fig 1A). From -10 to 0 minutes, participants orally swished the stimulus. Participants rinsed their mouths and gargled with 25 mL of the stimulus for 20 seconds. After 20 seconds, participants expectorated the sample and began rinsing with another 25 mL of stimulus. This was repeated for 10 minutes. This oral rinsing and expectorating phase of the test was conducted to maximize our chances of seeing pre-absorptive metabolic effects. At 0 minutes, participants ingested 300 mL of stimulus. Participants consumed the stimulus in less than 3 minutes. Blood samples were collected at 3, 6, 9, 12, 15, 30, 45, 60, 75, and 90 minutes.

10 participants were randomized in a crossover design to consume either a glucose solution (1.39 M glucose in 300 mL water) followed by continuous water ingestion or a glucose solution with added lactisole (1.39 M glucose + 2 mM lactisole dissolved in 300 mL water) followed by continuous 2 mM lactisole sipping (Fig 1D). Participants completed this test with the other solution at least one week later. This procedure was repeated in the reverse order following an ABBA design. Lactisole is an inverse agonist of the TAS1R2-TAS1R3 sweet taste receptor [33], and sipping throughout the OGTT was intended to prevent rebound receptor activity. From -10 to 0 minutes, participants orally swished the stimulus (Fig 1C). At 0 minutes, participants ingested 300 mL of stimulus. From 3 to 120 minutes, participants ingested water or 2 mM lactisole at a rate of 10 mL/min. Blood samples were collected at 3, 6, 9, 12, 15, 30, 45, 60, 75, 90, 105, and 120 minutes.

### Psychophysical studies

Psychophysical studies were conducted one week after completion of the oral glucose tolerance studies. We wished to characterize the sensitivities of participants to the sucralose and lactisole that were added to the OGTTs. 11 of the 12 participants who completed the glucose with sucralose tolerance study were asked to rate the taste intensity of six concentrations of sucralose. Participants were instructed not to consume food or drink other than water for at least one hour prior to testing. All testing occurred between the hours 9 am and noon. Participants were trained in the use of a general Labeled Magnitude Scale (gLMS). In each test session, samples were randomized and presented in 25 mL aliquots. Participants held each solution in the mouth for 5 seconds and rated the taste quality and intensity using a gLMS. Participants then expectorated and rinsed their mouths with water four times (Millipore). The interstimulus interval was 2 minutes. Each solution was tested by each participant in quadruplicate.

In a sweetness intensity study, participants rated perceived sweetness intensity of glucose and sucralose and the perceived salty intensity of sodium chloride (NaCl) (Sigma) on a gLMS. Glucose was presented in five half-log concentration steps ranging from 31.6 to 3160 mM (31.6, 100, 316, 1000, 3160 mM). Sucralose was presented in six half-log concentration steps ranging from 0.016 to 5 mM (0.0158, 0.05, 0.1806, 0.5, 1.581, 5 mM). Participants were also asked to rate the sweetness intensity of the glucose-sucralose mixture used in the glucose tolerance test. NaCl was tested as a negative control for bias in gLMS scale or number use. NaCl was presented in six half-log concentration steps ranging from 4 to 1270 mM (4, 12.7, 40, 127, 400, 1270 mM).

In a sweetness inhibition study, 7 of the 10 participants who completed the glucose with lactisole tolerance study rated on a gLMS the perceived sweetness intensity of 1.39 M glucose prepared in mixture with increasing concentrations of lactisole (0, 1, 2, 4, 8, and 16 mM) until the perceived sweetness intensity of 1.39 M glucose was reduced to ‘barely detectable’ intensity for each participant. We determined the concentration of lactisole that reduced sweetness ratings to <10% of their original rating of 1.39 M glucose.

### Statistical analyses

Repeated measures two-way ANOVA was used to determine whether plasma glucose, insulin, and glucagon responses differed between treatments. Post-hoc Bonferroni tests were used to assess pairwise differences at each time point and to correct for multiple comparisons. Paired T-tests were used to determine whether area under the curve (AUC) and peak concentrations differed between treatments. AUC was calculated using the trapezoidal method. Peak concentrations were determined for each participant in each test session. Differences in plasma responses between treatments were expressed as percentage changes from the control treatment (1.39 M glucose). Relationships between plasma responses fasting glucose, and taste ratings were assessed using Pearson’s correlations. Analyses were conducted using GraphPad (GraphPad Software, San Diego, CA). Statistical significance for alpha (Type 1 errors) was set at p < 0.05. The relationship between physiological and psychophysical responses to sucralose was assessed using Pearson’s correlations.

## Results

### Sucralose enhances plasma insulin responses to glucose consumption

**Fig 2:**
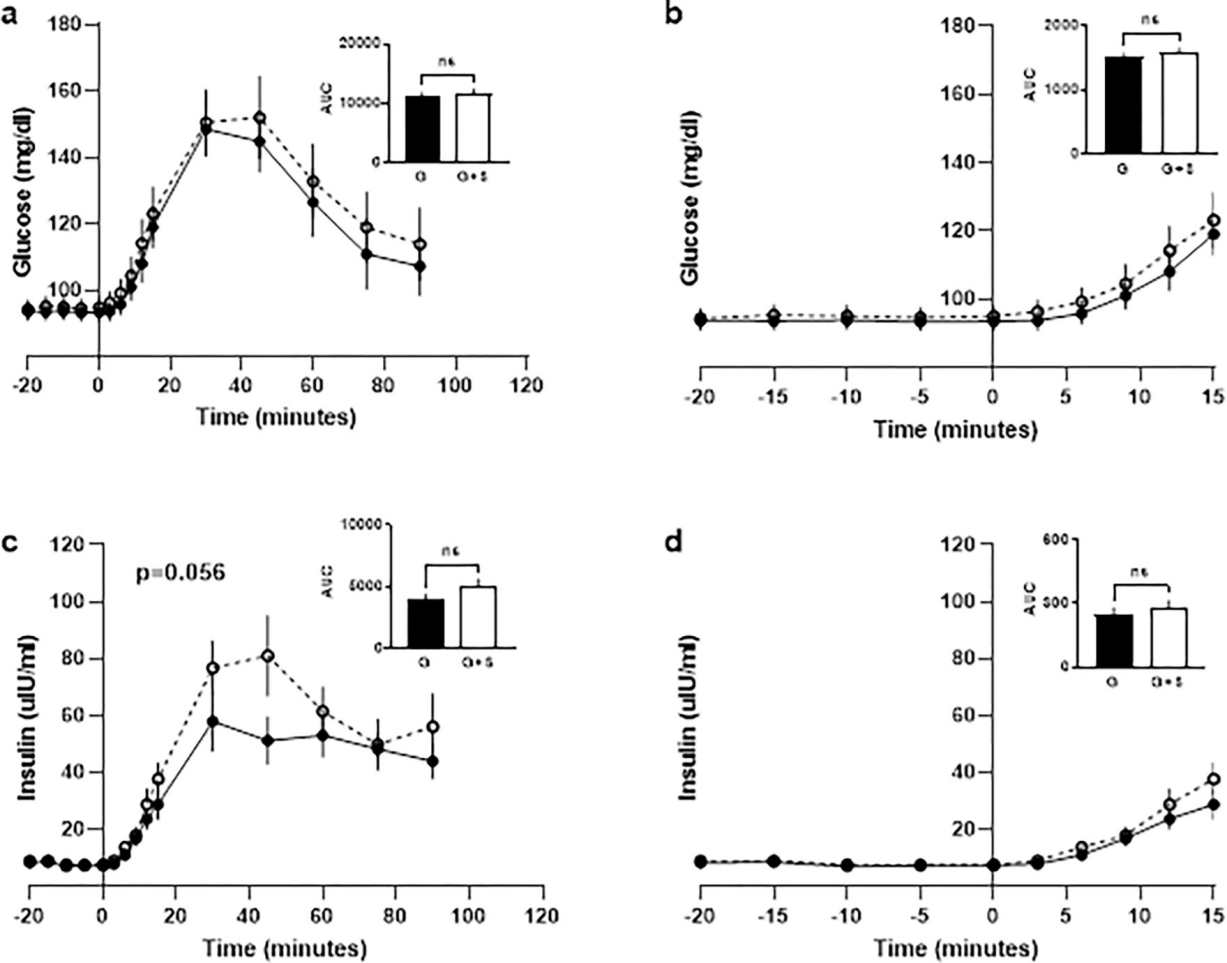


**Fig 3:**
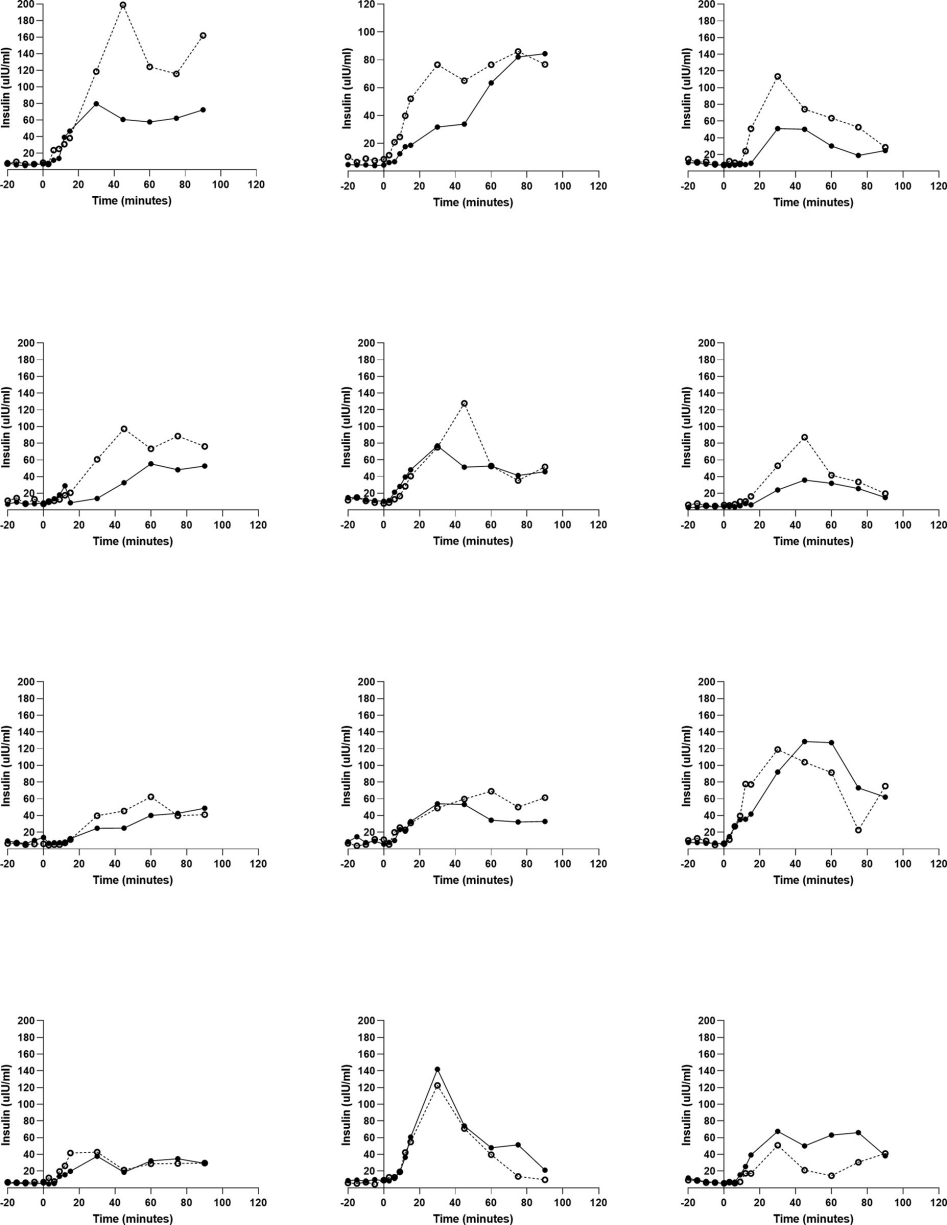


To determine whether TAS1R2-TAS1R3 hyperstimulation influences glucose tolerance, we conducted oral glucose tolerance tests in which participants consumed either 1.39 M glucose neat or 1.39 M glucose + 5 mM sucralose in a crossover design (Fig 1). There was a significant, positive correlation between insulin AUC from the first and second test sessions with the glucose OGTT, establishing test-retest reliability (R2 = 0.724, p<0.01, n = 12) (S1 Fig). Sucralose had no significant effects on plasma glucose, but significantly increased insulin (F(1, 10) = 4.55, p = 0.056, two-way ANOVA with the Greenhouse–Geisser correction for sphericity) (Fig 2A and 2C). There were no significant differences in plasma glucose or insulin AUC (paired Student’s T-test) and no differences in early responses in the first 0 to 15 minutes (Fig 2B and 2D). Plasma glucagon and plasma glucagon-like peptide-1 (GLP-1) were unaffected by the addition of sucralose to the OGTT (S2 and S4 Figs). Data from all 12 participants are displayed individually in separate panels of Fig 3. Adding sucralose to the OGTT elevated and/or left shifted insulin functions in 10 of the 12 participants.

### Perceived sucralose sweetness was correlated with increased plasma glucose and insulin when sucralose was added to OGTTs

**Fig 4:**
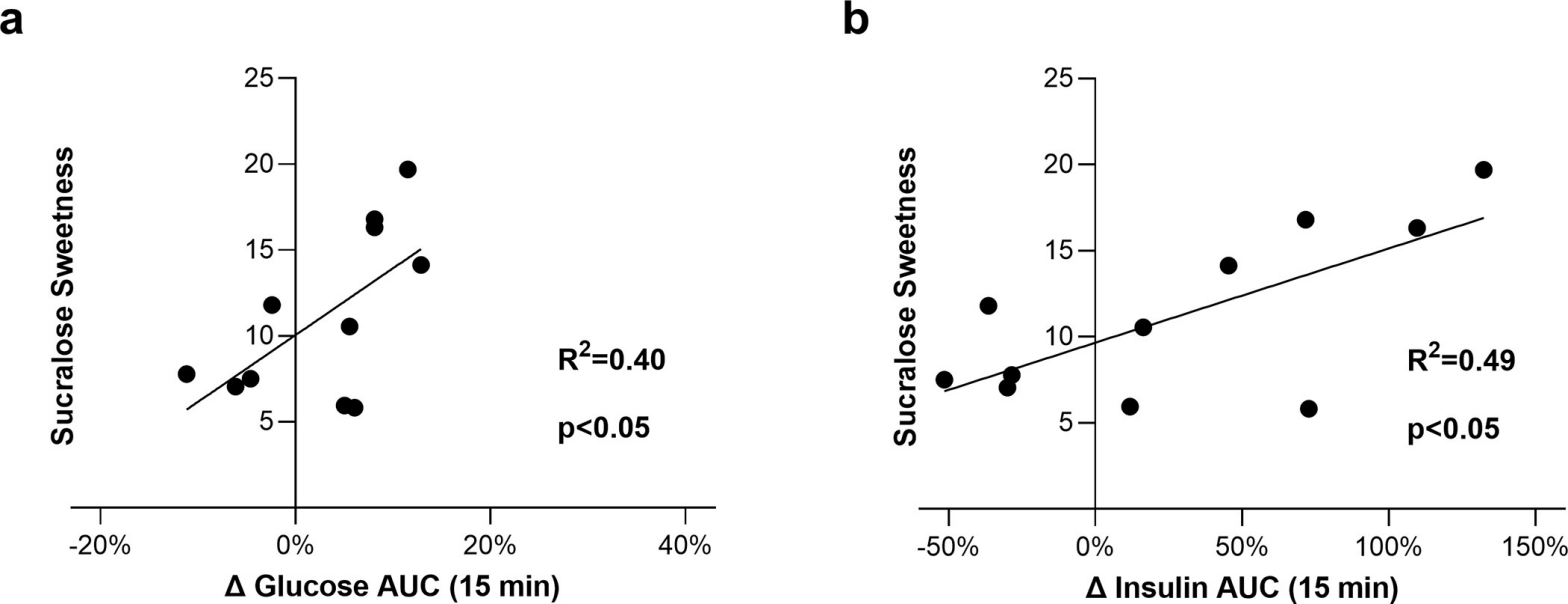


We evaluated sweet taste perception in participants in order to assess the relationship between the perception of sucralose (and thus stronger activation of TAS1R2-TAS1R3) and the effects of added sucralose on glucose metabolism. To measure sweet taste perception of sucralose, we asked participants to rate the perceived sweetness intensity elicited by six concentrations of sucralose. Delta plasma glucose or insulin for the first 15 minutes was calculated with the following formula: [(G+S)-(G)]/(G) x 100. Average sweetness ratings were positively correlated with early delta plasma glucose 15 minute AUC (R^2^ = 0.40, p<0.05) (Fig 4A), and early delta plasma insulin 15 minute AUC (R^2^ = 0.49, p<0.05) (Fig 4B). Overall AUCs were not correlated with sweetness ratings. Data were analyzed by linear regression. Those who perceived sucralose as sweetest tended to have the greatest increases in plasma glucose and insulin when sucralose was added to their OGTT.

To control for bias in scale or number use on the gLMS, average sucralose sweetness rating was correlated with average NaCl saltiness rating, revealing no relationship (R^2^ 0.06, p = 0.46) (S6 Fig). This indicates that individual differences are not driven by general oral sensitivity or trends in number use. We also analyzed the correlation of NaCl saltiness ratings versus plasma insulin AUC as we did with sucralose ratings and there was no relationship (data not shown).

### Lactisole added to ingested glucose did not significantly alter plasma glucose and insulin responses overall

**Fig 5:**
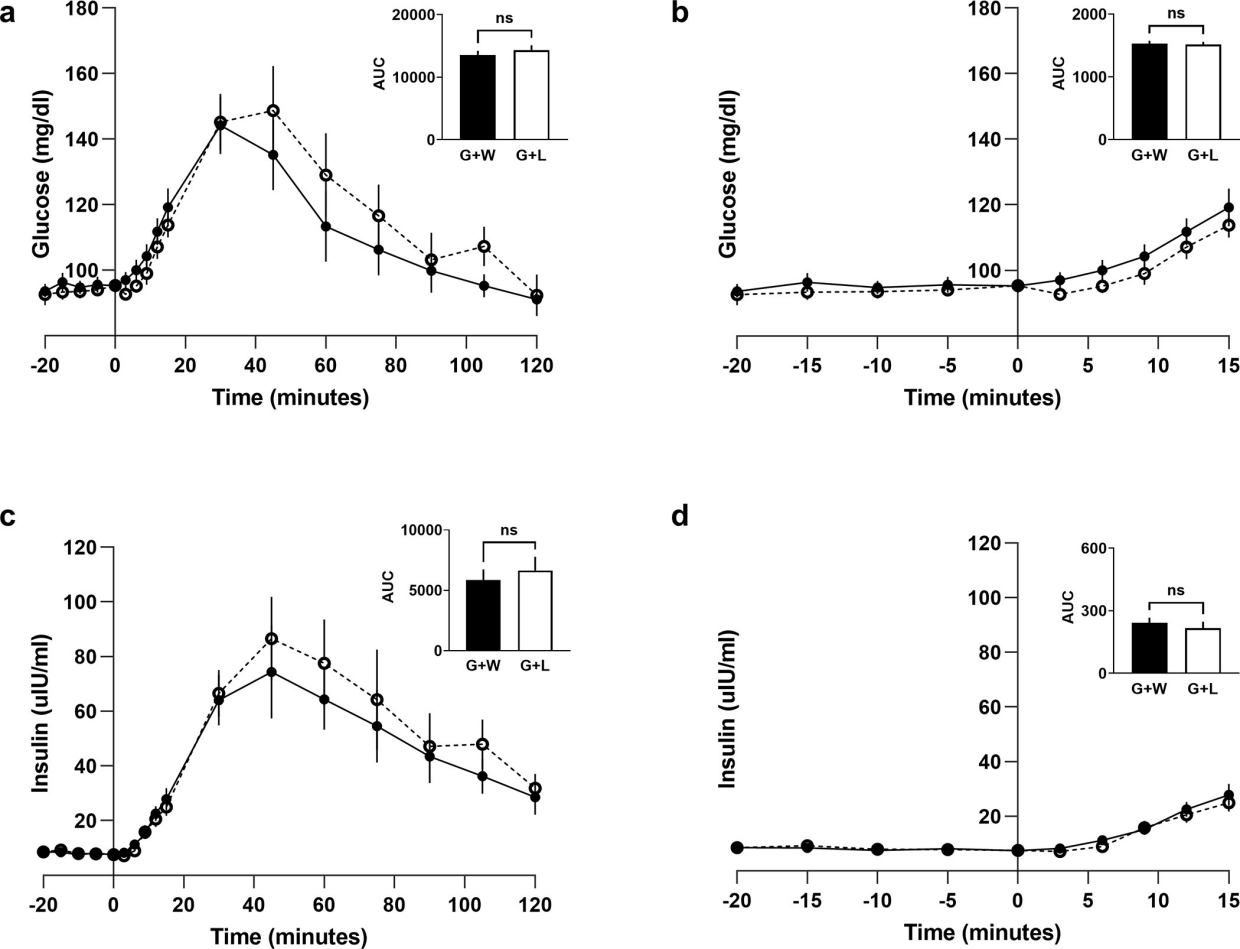


To determine whether TAS1R2-TAS1R3 inhibition influences glucose tolerance, we conducted oral glucose tolerance tests in which participants consumed either 1.39 M glucose + water or 1.39 M glucose + 2 mM lactisole in a crossover design. Lactisole had no effects on plasma glucose or insulin (two-way ANOVA) (Fig 5A–5D) and no effects on plasma glucagon or GLP-1 (S3 and S5 Figs).

### Sensitivity to lactisole was correlated with decreased plasma glucose and insulin when lactisole was added to OGTTs

**Fig 6:**
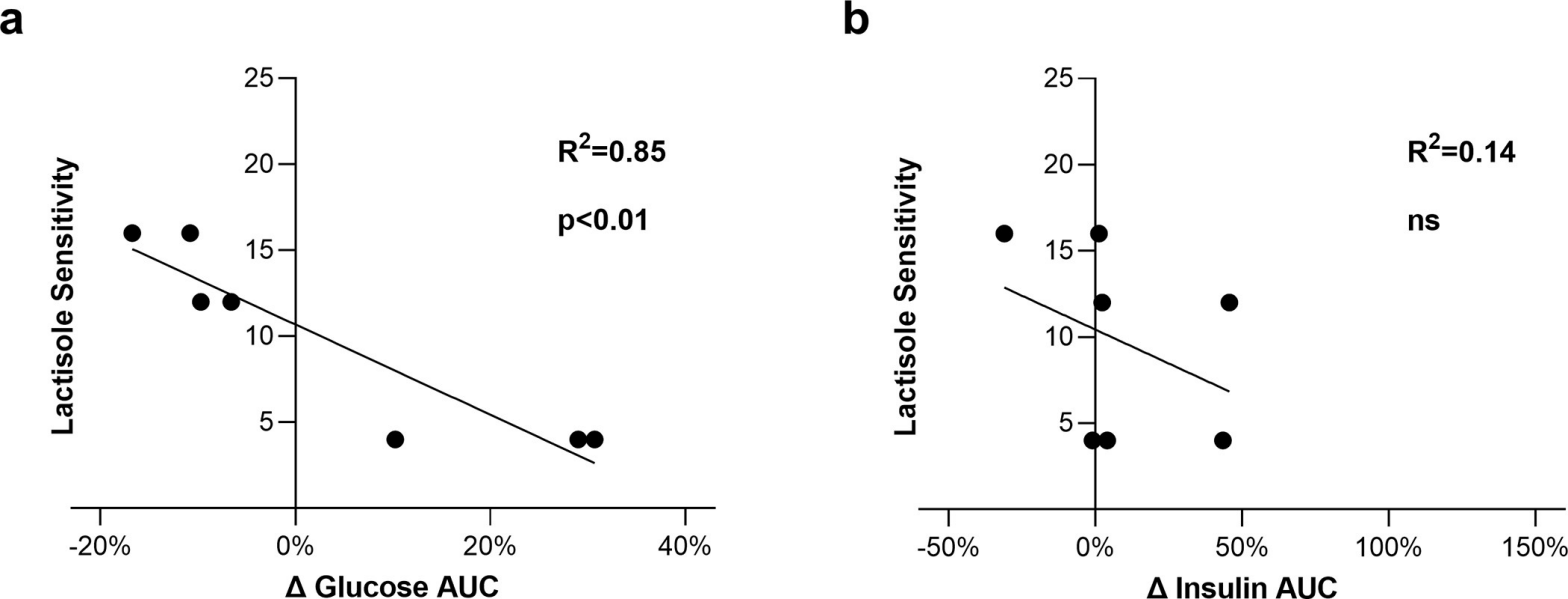


We evaluated lactisole sensitivity of participants by presenting 1.39 M glucose in mixture with increasing amounts of lactisole until perceived sweetness was reduced to ‘barely detectable’ intensity. Lactisole sensitivity is reported as mM of Na-lactisole required to inhibit sweetness on the y-axis. Lower numbers represent more sensitivity to lactisole and higher numbers represent less sensitivity. Delta plasma glucose and insulin was calculated with the formula: [(G+W)-(G+L)]/(G+W) x 100. Lactisole sensitivity was correlated with delta plasma glucose AUC for 120 minutes (R^2^ = 0.85, p<0.01) (Fig 6A), but not delta plasma insulin AUC (Fig 6B). Those who were most sensitive to lactisole (low on the y-axis) tended to have lower plasma glucose when lactisole was added to their OGTT, [(G+W)-(G+L)]/(G+W) x 100.

## Discussion

The purpose of these experiments was to determine whether TAS1R2-TAS1R3 participates in glucose regulation in humans. Here we show using a pharmacological push-pull (excitation and inhibition) approach that ingestion of either a TAS1R2-TAS1R3 agonist (sucralose) or an antagonist (lactisole) in mixture with glucose acutely altered human glucose tolerance in different ways consistent with our hypothesis. Ingestion of a glucose load with 5 mM sucralose enhanced plasma insulin peak (Fig 2C) in 10 out of 12 participants (Fig 3). Our hypothesis was wrong, however, in that it predicted an elevation in plasma glucose from added sucralose in the sample population. Sucralose sweetness ratings were correlated with both early increases in plasma glucose (R^2^ = 0.41, p<0.05) as well as increases in plasma insulin (R^2^ = 0.38, p<0.05) when sucralose was added to the OGTT (15 minute AUC) (Fig 4). This suggests that individual differences in TAS1R2-TAS1R3 function (as indicated by individual perceived sweetness intensity) lead to differences in glucose metabolism. These observations are consistent with studies that demonstrate TAS1R2-TAS1R3 stimulation promotes glucose transporter activity, glucose absorption [4,5], and early insulin responses [18]. Clearly some individuals have very large metabolic responses to sucralose when in mixture with glucose (see Fig 3). One possible explanation is due to variability in the TAS1R family of receptors [34], and in the gustducin gene *GNAT3*, a GTP-binding protein associated with TAS1R2-TAS1R3 transduction [35]. Given that this receptor and it’s transduction cascade are expressed in the intestine, pancreas, adipocytes, and elsewhere, high sensitivity to stimulation may influence physiological responses and predispose people to metabolic disease as a function of use of sugar and HPS in the diet [36].

In comparison, when TAS1R2-TAS1R3 activity was inhibited with 2 mM lactisole, there were no significant effects on plasma glucose or insulin overall, although there were trends for plasma glucose to be suppressed early and elevated later in the OGTT, as well as insulin to be elevated (Fig 5), which is consistent with our hypothesis and results of others [20,37]. Sensitivity to lactisole sweetness inhibition was correlated with decreased plasma glucose (R^2^ = 0.84, p<0.01) when lactisole was added to the OGTT over the whole test (120 minute AUC) (Fig 6). Those with the greatest sensitivity to lactisole’s inhibition of sweetness, showed the greatest reductions in plasma glucose during the OGTT. In support of this idea, TAS1R2 ablation in mice has been shown to reduce glucose absorption and total glucose AUC [6,18].

We provide evidence that TAS1R2-TAS1R3 activation facilitates glucose plasma regulation. Sucralose added to glucose significantly elevated plasma insulin peak (Fig 2C), likely due to facilitation of insulin secretion through oral, intestinal or pancreatic activation of TAS1R2-TAS1R3. Previous studies demonstrated that oral stimulation with glucose and glucose polymers, but not HPS, can elicit pre-absorptive insulin secretion [1,38–40]. Interestingly, preexposure to seven beverages containing a mixture of sucralose and maltodextrin over two weeks induces physiological changes that result in elevated insulin responses to a plain glucose OGTT [41] cf [42–47]. This observation suggests that not only does sucralose alter OGTT responses when in admixture with glucose but may have implications for acquired responses.

Glucose absorption rates could be increased during TAS1R2-TAS1R3 hyperstimulation due to the translocation of glucose transporters, which has been previously reported to occur in minutes [5,6]. In line with this, our results demonstrate that those who tended to perceive sucralose as sweetest had the largest increases in plasma glucose when sucralose was added to the OGTT (R^2^ = 0.40, p<0.05), and the largest increases in plasma insulin (R^2^ = 0.49, p<0.05) (Fig 4). Insulin may be elevated because sucralose directly stimulated pancreatic TAS1R2-TAS1R3. To this point, *in vitro*, sucralose and other HPS stimulate insulin secretion from cultured pancreatic beta cells [48–50]. When humans ingest sucralose, approximately 15% is absorbed by the intestine and enters circulation temporarily [51]. In the present study, we administered a relatively high dose of sucralose (600 mg), which makes pancreatic involvement more likely.

The TAS1R2-TAS1R3 inhibitor lactisole reduced plasma glucose AUC following an OGTT for those who were more lactisole-sensitive (R^2^ = 0.84, p<0.01). Lactisole is 99% absorbed into blood and is excreted 90% intact [52], in contrast with sucralose, which is only 15% absorbed [51]. Lactisole may have prevented translocation of glucose transporters in the intestine that is stimulated by TAS1R2-TAS1R3. In mice, TAS1R2-TAS1R3 activation appears to increase translocation of GLUT2 to the apical membrane of enterocytes and increase intestinal absorption [6]. TAS1R2-knockout mice exhibit reduced plasma glucose levels following glucose tolerance tests [6,18]. Additionally, TAS1R2 ablation may confer health benefits in the long-term. When fed an obesogenic diet, TAS1R2 knockout animals are partially protected against weight gain, fat mass gain, and hyperinsulinemia [19]. This suggests a relationship between health status and TAS1R2-TAS1R3 activity. To that point, we recently showed that clofibric acid, a fibrate drug which reduces plasma lipids and glucose, inhibits sweet taste perception in humans via inhibition of TAS1R2-TAS1R3 [53]. Our present results suggest that TAS1R2-TAS1R3 inhibition exerts acute physiological and metabolic effects.

### Limitations and future directions

A limitation to interpretation of our data is the small sample size, 10 or 12 participants per study. Some of our participants have low sensitivity to sucralose and to lactisole and, not surprisingly, this dampened the influence of TAS1R2 and TAS1R3 on glucose metabolism. Although we intended to examine taste sensitivity to sucralose and lactisole as a correlate of physiology, it is clear from our results that future studies should prescreen participants for the extremes of high sensitivity and insensitivity to sucralose and lactisole to delineate the role of TAS1R2-TAS1R3 sensitivity in glucose metabolism within the population. Another limitation is that it is unclear whether the effects observed with added sucralose were based upon action of TAS1R2-TAS1R3 in oral taste tissue, intestine, pancreas, or other tissues. In future studies we can deliver the stimuli directly to the stomach or intestine and bypass the mouth. Nevertheless, we provide evidence that a non-caloric TAS1R2-TAS1R3 agonist, sucralose, increases insulin responses to ingested glucose. The magnitude of this response was not equal for all participants, but was directly proportional to how sweet they perceived sucralose to be (R^2^ = 0.38, p<0.05) (Figs 3 and 4). Additionally, a limitation of interpreting the effects of lactisole on glucose metabolism is tied to the weak potency of glucose as a TAS1R2-TAS1R3 agonist. Although we did see an impact on plasma glucose among participants who were most sensitive to lactisole, we did not see an overall effect on plasma glucose and insulin in the sample population. Logically, one would expect lactisole as a TAS1R2-TAS1R3 antagonist to have its greatest effects when the receptor is strongly stimulated, such as would occur with fructose or sucralose ingested with glucose, an approach that others have utilized [20].

The observation that sucralose added to glucose can have large metabolic effects may be directly relevant to natural stimuli such as fructose, which is gram for gram the most potent sugar agonist of TAS1R2-TAS1R3. Future studies should consider whether fructose acts like sucralose as a glucose metabolic regulator. Fructose ingestion together with oral glucose potentiates glucose-stimulated insulin secretion (GSIS) in a TAS1R2-dependent manner, despite the fact that fructose in isolation is minimally insulinogenic [10]. Thus, there may be a parallel between the metabolic effects of sucralose added to glucose in the present study and the effects of fructose ingested with glucose. Current day dietary habits of excessive ingestion of sucrose and high fructose corn syrup could hyperstimulate TAS1R2-TAS1R3 and contribute to metabolic syndrome and pre-diabetes.

Although we know that TAS1R2-TAS1R3 is present in the pancreas, we do not fully understand its utility for this organ. It is important that the pancreas both senses absolute plasma glucose concentrations, as well as detects changes to plasma glucose concentrations. In many sensory systems absolute assessments come from non-adapting systems and change detectors are typically adapting systems. Future research should determine whether the pancreas needs two sensory systems, the metabolic signaling pathway and the TAS1R2-TAS1R3 system, to accomplish both of these sensory assessments.

## Conclusions

When added to an OGTT, sucralose tended to increase plasma glucose and increased insulin peak. These increases were correlated with sucralose sweetness ratings. Conversely, lactisole tended to lower glucose AUC early in the OGTT. This reduction in plasma glucose was correlated with lactisole sensitivity. Thus, TAS1R2-TAS1R3 appears to participate in glucose metabolism in a push-pull manner when manipulated with a non-caloric agonist and an antagonist. This suggests potential long term metabolic health implications of TAS1R2-TAS1R3 responses to sugars and sweeteners. Future studies should examine the effects of TAS1R2-TAS1R3 stimulation and inhibition in populations with obesity or impaired fasting glucose.

## Supporting information

S1 Fig Scatterplot showing test-retest reliability of insulin response to repeated oral glucose tolerance tests. There was a significant, positive correlation between insulin AUC from the first and second test sessions with glucose (R^2^ = 0.724, p<0.01), n = 12. Data were analyzed by linear regression, R^2^.
(PDF)

S2 Fig Effects of sucralose on plasma glucagon. 12 healthy participants ingested either 1.39 M glucose (solid line) or 1.39 M glucose + 5 mM sucralose (dashed line). Blood samples were collected from baseline to 90 minutes after ingestion and analyzed for plasma glucagon. There was no significant difference in plasma glucagon response (two-way ANOVA).
(PDF)

S3 Fig Effects of lactisole on plasma glucagon. 10 healthy participants ingested either 1.39 M glucose + water (solid line) or 1.39 M glucose + 2 mM lactisole (dashed line). Blood samples were collected from baseline to 120 minutes after ingestion and analyzed for plasma glucagon. There was no significant difference in plasma glucagon response (two-way ANOVA).
(PDF)

S4 Fig Effects of sucralose on plasma glucagon-like peptide-1 (GLP-1). 12 healthy participants ingested either 1.39 M glucose (solid line) or 1.39 M glucose + 5 mM sucralose (dashed line). Blood samples were collected from baseline to 90 minutes after ingestion and analyzed for plasma GLP-1. There was no significant difference in plasma GLP-1 response (two-way ANOVA).
(PDF)

S5 Fig Effects of lactisole on plasma glucagon-like peptide-1 (GLP-1). 10 healthy participants ingested either 1.39 M glucose + water (solid line) or 1.39 M glucose + 2 mM lactisole (dashed line). Blood samples were collected from baseline to 120 minutes after ingestion and analyzed for plasma GLP-1. There was no significant difference in plasma GLP-1 response (two-way ANOVA).
(PDF)

S6 Fig Correlations of perceived sucralose sweetness with perceived sodium chloride (NaCl) saltiness. 11 of the 12 participants who completed the glucose with sucralose tolerance study were asked to rate the sweetness intensity of six concentrations of sucralose (0.016 to 5 mM) and the saltiness intensity of six concentrations of NaCl (4 to 1270 mM) in half-log steps using a general Labeled Magnitude Scale (gLMS) four times. There was no significant correlation between the mean sweetness ratings of sucralose and the mean saltiness ratings of NaCl (R^2^ = 0.06, p = 0.46). Data were analyzed by linear regression, R^2^.
(PDF)

S1 File Original Data File may be found with Supporting Information as: Kochem Plos One Data final. (XLSX)
