# Supplementary figures and images for: Activation and inhibition of the sweet taste receptor TAS1R2-TAS1R3 differentially affect glucose tolerance in humans
Source: PLoS One. 2024 May 1;19(5):e0298239. doi: 10.1371/journal.pone.0298239 (PMC11062524; doi:10.1371/journal.pone.0298239)

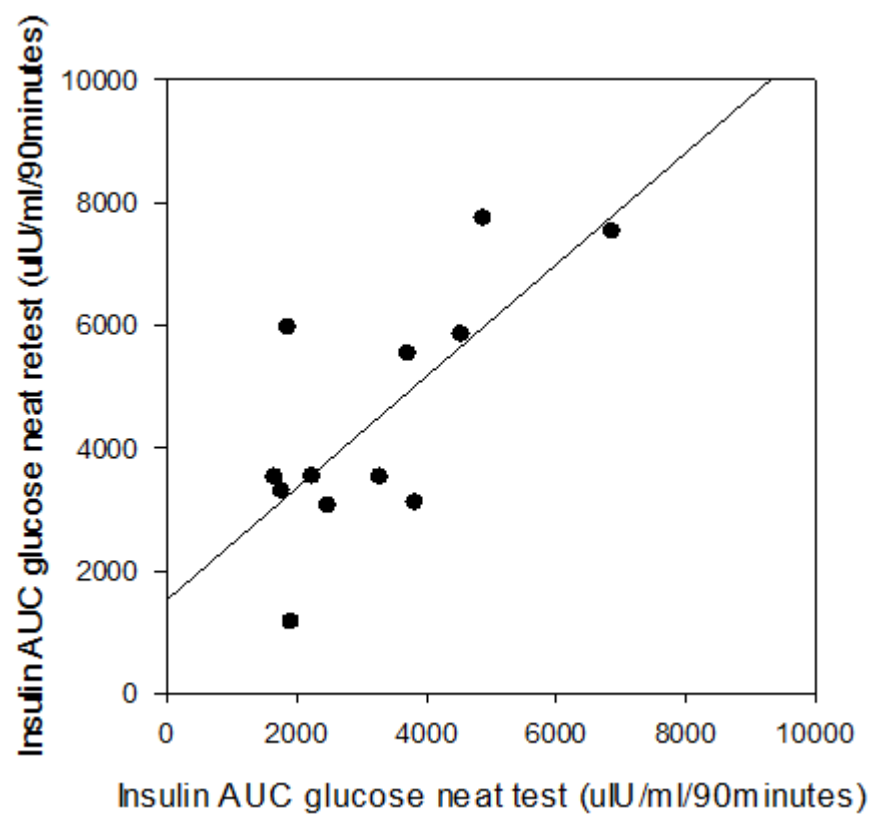

Supplement: S1 Fig — There was a significant, positive correlation between insulin AUC from the first and second test sessions with glucose (R2 = 0.724, p<0.01), n = 12. Data were analyzed by linear regression, R2. (PDF) [file pone.0298239.s001.pdf]

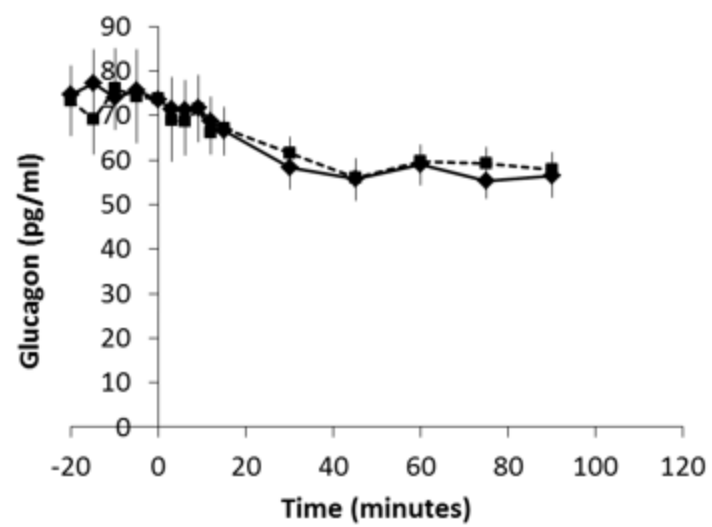

Supplement: S2 Fig — 12 healthy participants ingested either 1.39 M glucose (solid line) or 1.39 M glucose + 5 mM sucralose (dashed line). Blood samples were collected from baseline to 90 minutes after ingestion and analyzed for plasma glucagon. There was no significant difference in plasma glucagon response (two-way ANOVA). (PDF) [file pone.0298239.s002.pdf]

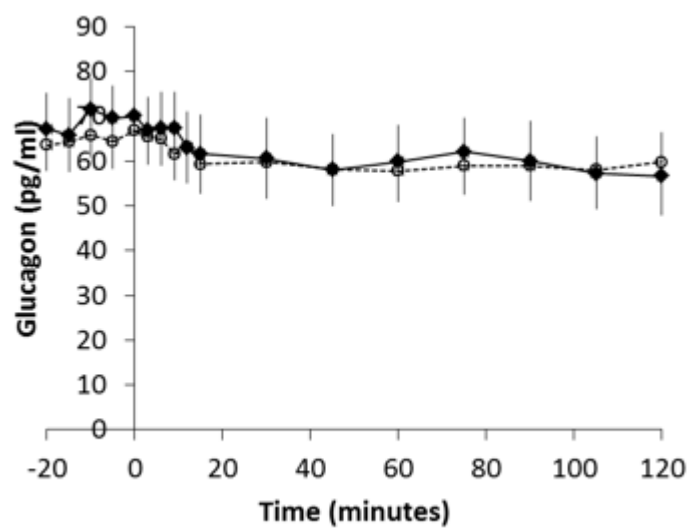

Supplement: S3 Fig — 10 healthy participants ingested either 1.39 M glucose + water (solid line) or 1.39 M glucose + 2 mM lactisole (dashed line). Blood samples were collected from baseline to 120 minutes after ingestion and analyzed for plasma glucagon. There was no significant difference in plasma glucagon response (two-way ANOVA). (PDF) [file pone.0298239.s003.pdf]

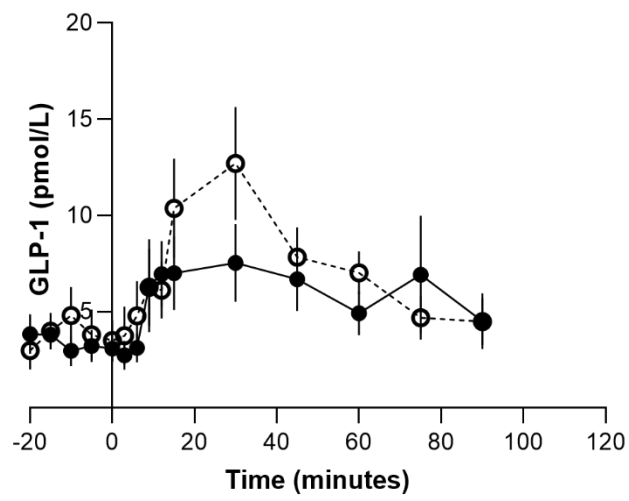

Supplement: S4 Fig — 12 healthy participants ingested either 1.39 M glucose (solid line) or 1.39 M glucose + 5 mM sucralose (dashed line). Blood samples were collected from baseline to 90 minutes after ingestion and analyzed for plasma GLP-1. There was no significant difference in plasma GLP-1 response (two-way ANOVA). (PDF) [file pone.0298239.s004.pdf]

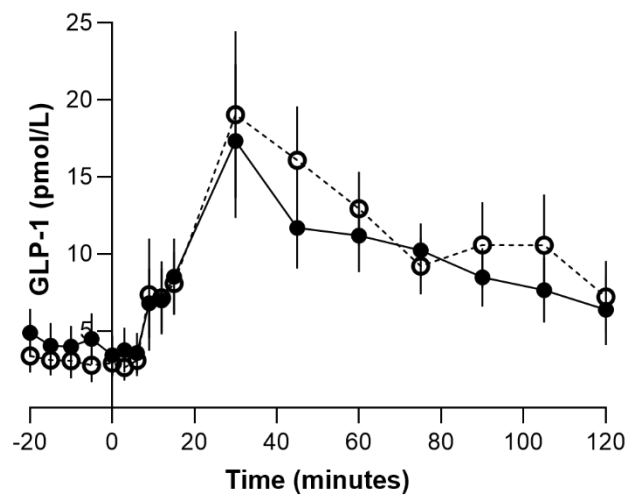

Supplement: S5 Fig — 10 healthy participants ingested either 1.39 M glucose + water (solid line) or 1.39 M glucose + 2 mM lactisole (dashed line). Blood samples were collected from baseline to 120 minutes after ingestion and analyzed for plasma GLP-1. There was no significant difference in plasma GLP-1 response (two-way ANOVA). (PDF) [file pone.0298239.s005.pdf]

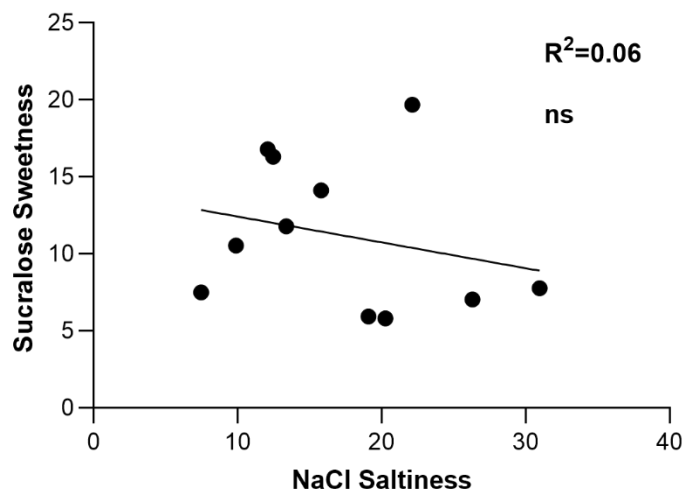

Supplement: S6 Fig — 11 of the 12 participants who completed the glucose with sucralose tolerance study were asked to rate the sweetness intensity of six concentrations of sucralose (0.016 to 5 mM) and the saltiness intensity of six concentrations of NaCl (4 to 1270 mM) in half-log steps using a general Labeled Magnitude Scale (gLMS) four times. There was no significant correlation between the mean sweetness ratings of sucralose and the mean saltiness ratings of NaCl (R2 = 0.06, p = 0.46). Data were analyzed by linear regression, R2. (PDF) [file pone.0298239.s006.pdf]
